# Prognostic Model Based on Systemic Inflammatory Response and Clinicopathological Factors to Predict Outcome of Patients with Node-Negative Gastric Cancer

**DOI:** 10.1371/journal.pone.0128540

**Published:** 2015-06-15

**Authors:** Jing-lei Qu, Xiu-juan Qu, Zhi Li, Jing-dong Zhang, Jing Liu, Yue-e Teng, Bo Jin, Ming-fang Zhao, Ping Yu, Jing Shi, Ling-yu Fu, Zhen-ning Wang, Yun-peng Liu

**Affiliations:** 1 Department of Medical Oncology, The First Hospital of China Medical University, Shenyang, Liaoning, China; 2 Department of Clinical Epidemiology and Evidence-based Medicine, The First Hospital of China Medical University, Shenyang, Liaoning, China; 3 Department of Surgical Oncology, The First Hospital of China Medical University, Shenyang, Liaoning, China; INRS, CANADA

## Abstract

Prognostic models are generally used to predict gastric cancer outcomes. However, no model combining patient-, tumor- and host-related factors has been established to predict outcomes after radical gastrectomy, especially outcomes of patients without nodal involvement. The aim of this study was to develop a prognostic model based on the systemic inflammatory response and clinicopathological factors of resectable gastric cancer and determine whether the model can improve prognostic accuracy in node-negative patients. We reviewed the clinical, laboratory, histopathological and survival data of 1397 patients who underwent radical gastrectomy between 2007 and 2013. Patients were split into development and validation sets of 1123 and 274 patients, respectively. Among all 1397 patients, 545 had node-negative gastric cancer; 440 were included in the development set, 105 were included in the validation set. A prognostic model was constructed from the development set. The scoring system was based on hazard ratios in a Cox proportional hazard model. In the multivariate analysis, age, tumor size, Lauren type, depth of invasion, lymph node metastasis, and the neutrophil—lymphocyte ratio were independent prognostic indicators of overall survival. A prognostic model was then established based on the significant factors. Patients were categorized into five groups according to their scores. The 3-year survival rates for the low- to high-risk groups were 98.9%, 92.8%, 82.4%, 58.4%, and 36.9%, respectively (*P* < 0.001). The prognostic model clearly discriminated patients with stage pT1-4N0M0 tumor into four risk groups with significant differences in the 3-year survival rates (*P* < 0.001). Compared with the pathological T stage, the model improved the predictive accuracy of the 3-year survival rate by 5% for node-negative patients. The prognostic scores also stratified the patients with stage pT4aN0M0 tumor into significantly different risk groups (*P* = 0.004). Furthermore, the predictive value of this model was validated in an independent set of 274 patients. This model, which included the systemic inflammatory markers and clinicopathological factors, is more effective in predicting the prognosis of node-negative gastric cancer than traditional staging systems. Patients in the high-risk group might be good candidates for adjuvant chemotherapy.

## Introduction

Both Eastern and Western countries have agreed that postoperative adjuvant chemotherapy can improve survival of patients with gastric cancer. A meta-analysis showed that chemotherapy resulted in a 15% reduction in the mortality hazard compared with surgery alone [1]. However, subgroup analysis showed chemotherapy was associated with a trend toward better survival in patients without nodal involvement, although without statistical significance. Later, the CLASSIC study showed that postoperative adjuvant chemotherapy did not improve the 3-year disease-free survival rate of patients with node-negative gastric cancer [2]. In contrast, the ACTS-GC study suggested that patients without nodal involvement benefit from postoperative adjuvant chemotherapy [3]. One cause of these inconsistent results might be the enrollment of patients with different recurrence risks. For patients without lymph node metastasis, the ones who can benefit from chemotherapy are limited, and most of them fall victims to chemotherapy. Many factors in addition to the TNM stage also affect patients’ outcomes, and adequate risk stratification by a single factor is difficult. Therefore, establishment of a prognostic model that integrates a variety of factors associated with survival is necessary to discriminate patients at high risk, and these patients may truly benefit from adjuvant therapy.

An ideal prognostic model should be objective, reliable, and clinically useful. Traditional TNM staging has generally been used to predict the prognosis of gastric cancer. However, we have occasionally encountered patients with early-stage tumor who experienced recurrence shortly after surgery [4]. Obviously, TNM staging alone cannot predict the risk of recurrence. Tumor progression is not only determined by the intrinsic properties of tumor cells, but also by the host’s reaction to the tumor [5,6]. The most widely used predictive models of malignancy are currently the international prognostic index for aggressive non-Hodgkin’s lymphoma and the follicular lymphoma international prognostic index [7,8]. These indices include patient- and tumor-related characteristics as well as the host’s reaction to the tumor. They can be used to categorize patients into distinct prognostic groups, and the corresponding treatment strategies were also different. This highlights the idea of using a combination of clinically available patient-, tumor- and host-related factors to assess the prognosis and improve treatment choices. Recent studies have suggested that an index of the inflammatory response, which reflects the host’s reaction to tumor hypoxia, tissue injury, and necrosis, is associated with the prognosis of gastric cancer [9–11]. Although the prognostic factors of gastric cancer have been extensively described, no prognostic model based on the systemic inflammatory markers and clinicopathologic factors has been established to predict the survival of patients who have undergone radical gastrectomy, especially patients without nodal involvement.

This study was performed to construct a prognostic model incorporating the systemic inflammatory markers and clinicopathologic parameters of patients with resectable gastric cancer to identify patients at high risk. Furthermore, we evaluated whether the model can improve prognostic accuracy in node-negative patients, and suggested adjuvant therapy need to be considered for the high-risk patients.

## Patients and Methods

### Ethics statement

This retrospective study was approved by the institutional review board of the First Hospital of China Medical University. Written informed consent was obtained from each participant before enrollment.

### Patients

We retrospectively reviewed the data of 1598 patients who underwent gastrectomy and D2 lymphadenectomy from January 2007 to December 2013 in the First Hospital of China Medical University. Of these 1598 patients, 1397 met the following eligibility criteria: (1) histologically confirmed stage I to III gastric cancer according to the seventh edition of the American Joint Committee on Cancer (AJCC) TNM Staging System [12]; (2) complete blood cell count with differential, plasma fibrinogen level, and serum albumin level measured within 7 days preoperatively; and (3) availability of complete follow-up data. The exclusion criteria were: (1) a history of double cancer, (2) neoadjuvant chemotherapy or adjuvant radiotherapy, (3) death within 3 months of surgery, and (4) clinical evidence of infection or other inflammatory conditions. Patients who underwent surgical resection of gastric cancer between December 2008 and December 2013 were assigned to a development set (n = 1123), and patients who underwent surgical resection between January 2007 and November 2008 were assigned to an independent validation set (n = 274). Of all included patients, 545 had histopathologically confirmed gastric cancer without lymph node involvement; 440 were included in the development set, 105 were included in the validation set.

### Blood sample analyses

Blood samples were taken for routine laboratory analysis before breakfast within 7 days preoperatively. The white blood cell count (reference range, 3.5–9.5 × 10^9^/L), neutrophil count (reference range, 1.8–6.3 × 10^9^/L), lymphocyte count (reference range, 1.1–3.2 × 10^9^/L), platelet count (reference range, 125–350 × 10^9^/L), and hemoglobin level (reference range, 115–150 g/L for females, 130–175 g/L for males) were analyzed with an automated hematological blood analyzer (Sysmex XE-5000; Sysmex Corporation, Kobe, Japan). Serum concentrations of albumin (reference range, 40–55 g/L) were measured with an autoanalyzer (Hitachi 7600–210; Hitachi Co., Tokyo, Japan). Plasma concentrations of fibrinogen (reference range, 2–4 g/L) were measured with another autoanalyzer (STA-R Evolution; Diagnostica Stago, Asnières sur Seine, France). The neutrophil—lymphocyte ratio (NLR) was calculated by dividing the absolute neutrophil count by the absolute lymphocyte count. The platelet—lymphocyte ratio (PLR) was calculated by dividing the absolute platelet count by the absolute lymphocyte count.

### Statistical analysis

The prognostic model was developed using the development set. The primary analysis of the study was overall survival (OS), which was measured from the time of surgery to the time of death or the last follow-up visit. Chi-square tests were used to determine the significance of differences between development and validation sets. The survival curves were created by the Kaplan—Meier method, and differences between the curves were assessed by the two-tailed log-rank test. Univariate and multivariate analyses using a Cox proportional hazard model were carried out to access the relationship of systemic inflammatory markers and clinicopathologic parameters with OS. All significant factors in the univariate analysis were entered into a multivariate analysis using the forward stepwise (likelihood ratio) method. A prognostic model was established by all factors found to be significantly associated with survival in the multivariate analysis. The hazard ratios (HRs) were used to derive weighting factors of each prognostic factor to assess the differential risks of mortality. Coefficients were calculated by dividing the HRs of each prognostic factor by the smallest one (1.345) and rounding the resulting ratios to the nearest integer value [13]. Every patient was then assigned a prognostic index, which was derived by summing the coefficient of each significant prognostic factor in the final model. Two-sided *P* values of <0.05 were considered statistically significant for all tests. Statistical analysis was performed using SPSS 19.0 (IBM Corp., Armonk, NY, USA). The prognostic accuracy of the model was determined by receiver operating characteristic (ROC) analysis.

## Results

### Patient characteristics

A total of 1123 patients were assigned to the development set in this study (Table 1). The patients comprised 802 men and 321 women. The median age was 59 years (range, 25–85 years). The median tumor size was 4.5 cm (range, 0.3–18.0 cm). Fifty percent (567 of 1123) of the patients had T4 stage tumor. Thirty-nine percent (440 of 1123) of the patients had no lymph node involvement, among which 102 patients had T4a tumors. The median follow-up time was 27 months (range, 4–67 months). A total of 274 patients were assigned to the validation set (Table 1). When we compared the characteristics of the patients in the development and validation sets, we found no significant differences between these two groups (Table 1).

**Table 1:** Patient characteristics of the development and validation set.

Characteristics	Development set	Validation set	P value
	(n = 1123)	(n = 274)	
	No of patients (%)	No of patients (%)	
Age at diagnosis (median, range)	59 (25–85)	58 (26–82)	
< 65	763 (67.9)	186 (67.9)	
≥ 65	360 (32.1)	88 (32.1)	0.985
Gender			
Male	802 (71.4)	199 (72.6)	
Female	321 (28.6)	75 (27.4)	0.690
Tumor size (median, range)	4.5 (0.3–18.0)	4.0 (0.5–14.5)	
≤ 4.5 cm	605 (53.9)	157 (57.3)	
> 4.5 cm	518 (46.1)	117 (42.7)	0.307
Tumor location			
Lower 1/3	690 (61.4)	171 (62.4)	
Middle 1/3	185 (16.5)	50 (18.2)	
Upper 1/3	51 (4.5)	11 (4.0)	
2/3 or more	197 (17.5)	42 (15.3)	0.750
Lauren type			
Intestinal	453 (40.3)	122 (44.5)	
Diffuse	436 (38.8)	106 (38.7)	
Mixed	234 (20.8)	46 (16.8)	0.254
Depth of invasion			
T1	232 (20.7)	56 (20.4)	
T2	159 (14.2)	41 (15.0)	
T3	165 (14.7)	44 (16.1)	
T4	567 (50.5)	133 (48.5)	0.909
Metastatic LNs, No.			
0	440 (39.2)	105 (38.3)	
1–2	199 (17.7)	37 (13.5)	
3–6	194 (17.3)	51 (18.6)	
7–15	175 (15.6)	51 (18.6)	
>15	115 (10.2)	30 (10.9)	0.414
Histology grade			
G1-G2	295 (26.3)	73 (26.7)	
G3-G4	828 (73.7)	200 (73.0)	0.874
Lymphovascular invasion			
Negative	792 (70.5)	198 (72.3)	
Positive	331 (29.5)	76 (27.8)	0.570
WBC count (×10^3^ mm^-3^)			
≤ 9.5	1022 (91.0)	249 (90.9)	
> 9.5	101 (9.0)	25 (9.1)	0.946
Neutrophil count (×10^3^ mm^-3^)			
≤ 6.3	1012 (90.1)	248 (90.5)	
> 6.3	111 (9.9)	26 (9.5)	0.844
Lymophocyte count (×10^3^ mm^-3^)			
< 1.1	138 (12.3)	24 (8.8)	
≥ 1.1	985 (87.7)	250 (91.2)	0.102
Platelet count (×10^3^ mm^-4^)			
≤ 350	1045 (93.1)	248 (90.5)	
> 350	78 (6.9)	26 ((9.5)	0.150
NLR			
≤ 1.86	562 (50.0)	143 (52.2)	
> 1.86	561 (50.0)	131 (47.8)	0.524
PLR			
≤ 168	843 (75.0)	210 (76.6)	
> 168	280 (24.9)	64 (23.4)	0.587
Hemoglobin (g/l)			
< 115	312 (27.8)	66 (24.1)	
≥ 115	811 (72.2)	208 (75.9)	0.217
Serum albumin (g/l)			
< 40	482 (42.9)	116 (42.3)	
≥ 40	641 (57.1)	158 (57.7)	0.861
Plasma fibrinogen (mg/dl)			
≤ 400	555 (49.4)	143 (52.2)	
> 400	568 (50.6)	131 (47.8)	0.411

Abbreviations: LNs, lymph nodes; G1, well differentiated; G2, moderately differentiated; G3, poorly differentiated; G4, undifferentiated; WBC, white blood cells; NLR, neutrophil—lymphocyte ratio; PLR, platelet—lymphocyte ratio; HR, hazard ratio; CI, confidence interval.

### NLR and PLR cutoffs

The patients in the development set were divided into equal quartiles according to the NLR and PLR. The 25th, 50th, and 75th NLR percentiles were 1.41, 1.86, and 2.73, respectively. The 25th, 50th, and 75th PLR percentiles were 91, 121, and 168, respectively. We then used Cox regression to examine the association of the NLR and PLR quartiles with survival. The HRs for the second, third, and fourth NLR quartiles compared with the first quartile were 1.33 (*P* = 0.135), 1.71 (*P* = 0.003), and 2.13 (*P* < 0.001), respectively. The HRs for the second, third, and fourth PLR quartiles compared with the first quartile were 1.04 (*P* = 0.843), 1.38 (*P* = 0.073), and 1.99 (*P* < 0.001), respectively. Based on these results, we decided to use the 50th NLR and 75th PLR percentiles as cutoff values to predict patients’ prognoses.

### Analysis of independent prognostic factors

The relationship of clinicopathological characteristics and systemic inflammation markers with OS in patients of the development set is shown in Table 2. With respect to clinicopathologic parameters, univariate analysis demonstrated that age, tumor size, tumor location, Lauren type, depth of invasion, lymph node metastasis, histological grade, and lymphovascular invasion had prognostic significance. With respect to systemic inflammation markers, a higher NLR, PLR, and fibrinogen level and lower lymphocyte, hemoglobin, and albumin level were associated with a higher risk of mortality. In the multivariate analysis, age, tumor size, Lauren type, depth of invasion, lymph node metastasis, and NLR were identified as independent predictors of OS (Table 3).

**Table 2:** Univariate analysis for overall survival in the development set.

Characteristics	HR	95% CI	P value
Age at diagnosis (median, range)			
< 65	1.000		
≥ 65	1.597	1.258–2.028	< 0.001
Gender			
Male	1.000		
Female	1.171	0.909–1.510	0.222
Tumor size (median, range)			
≤ 4.5 cm	1.000		
> 4.5 cm	2.725	2.119–3.505	< 0.001
Tumor location			
Lower 1/3	1.000		
Middle 1/3	1.236	0.876–1.745	0.228
Upper 1/3	1.730	1.046–2.861	0.033
2/3 or more	2.070	1.568–2.733	< 0.001
Lauren type			
Intestinal	1.000		
Diffuse	1.657	1.262–2.176	< 0.001
Mixed	1.706	1.228–2.370	0.001
Depth of invasion			
T1	1.000		
T2	6.344	2.402–16.753	< 0.001
T3	13.316	5.274–33.619	< 0.001
T4	21.167	8.715–51.409	< 0.001
Metastatic LNs, No.			
0	1.000		
1–2	3.050	1.942–4.789	< 0.001
3–6	4.140	2.685–6.385	< 0.001
7–15	7.955	5.300–11.939	< 0.001
>15	10.688	7.030–16.248	< 0.001
Histology grade			
G1-G2	1.000		
G3-G4	1.361	1.024–1.809	0.033
Lymphovascular invasion			
Negative	1.000		
Positive	2.549	2.014–3.227	< 0.001
WBC count (×10^3^ mm^-3^)			
≤ 9.5	1.000		
> 9.5	1.440	0.991–2.093	0.056
Neutrophil count (×10^3^ mm^-3^)			
≤ 6.3	1.000		
> 6.3	1.438	0.999–2.069	0.050
Lymophocyte count (×10^3^ mm^-3^)			
< 1.1	1.000		
≥ 1.1	0.611	0.441–0.846	0.003
Platelet count (×10^3^ mm^-4^)			
≤ 350	1.000		
> 350	1.222	0.797–1.873	0.357
NLR			
≤ 50th percentile	1.000		
> 50th percentile	1.648	1.296–2.095	< 0.001
PLR			
≤ 75th percentile	1.000		
> 75th percentile	1.762	1.372–2.264	< 0.001
Hemoglobin (g/l)			
< 115	1.000		
≥ 115	0.713	0.557–0.913	0.007
Serum albumin (g/l)			
< 40	1.000		
≥ 40	0.674	0.533–0.853	0.001
Plasma fibrinogen (mg/dl)			
≤ 400	1.000		
> 400	1.724	1.351–2.200	< 0.001

Abbreviations: LNs, lymph nodes; G1, well differentiated; G2, moderately differentiated; G3, poorly differentiated; G4, undifferentiated; WBC, white blood cells; NLR, neutrophil—lymphocyte ratio; PLR, platelet—lymphocyte ratio; HR, hazard ratio; CI, confidence interval.

**Table 3:** Multivariate analysis for overall survival in the development set and prognostic score of patients with gastric cancer.

Variables	HR	95% CI	*P* value	Score
Age at diagnosis (years)				
< 65	1.000			0
≥ 65	1.445	1.129–1.850	0.003	1
Tumor size				
≤ 4.5 cm	1.000			0
> 4.5 cm	1.345	1.030–1.756	0.030	1
Lauren type				
Intestinal	1.000			0
Diffuse	1.594	1.204–2.112	0.001	1
Mixed	1.435	1.026–2.006	0.035	1
Depth of invasion				
T1	1.000			0
T2	4.136	1.537–11.126	0.005	3
T3	6.453	2.474–16.830	< 0.001	5
T4	7.567	2.974–19.253	< 0.001	6
Metastatic LNs, No.				
0	1.000			0
1–2	1.675	1.053–2.667	0.030	1
3–6	2.173	1.387–3.404	0.001	2
7–15	3.774	2.456–5.799	< 0.001	3
>15	4.488	2.862–7.039	< 0.001	3
NLR				
≤ 1.86	1.000			0
> 1.86	1.379	1.082–1.758	0.009	1

Abbreviations: LNs, lymph nodes; NLR, neutrophil—lymphocyte ratio; HR, hazard ratio; CI, confidence interval.

### Prognostic model and risk groups

**Fig 1:**
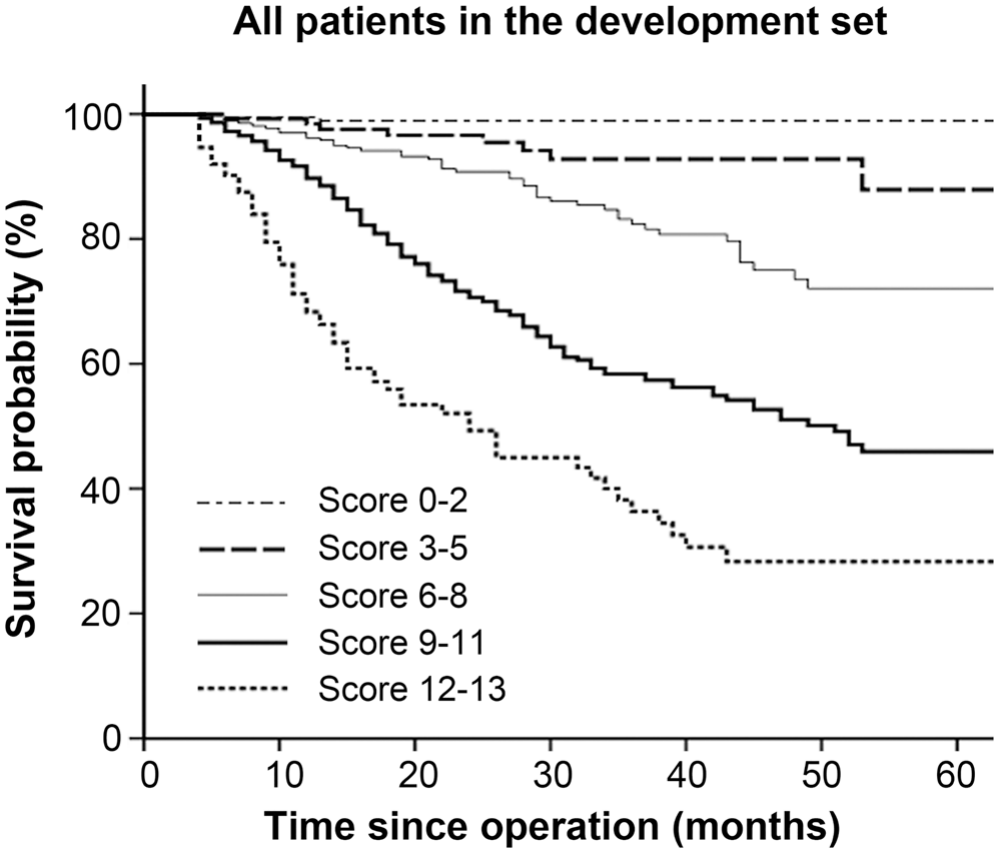
Survival curves based on risk groups for all patients who underwent gastrectomy and D2 lymphadenectomy in the development set (n = 1123).

In the development set of 1123 patients, the prognostic model was constructed using the statistically significant prognostic factors obtained in the multivariate analysis. Table 3 shows the scores based on the HRs in the Cox hazard model; a prognostic index score was then developed for each patient. According to the cutoffs chosen at approximately equal distance along the range of scores, patients with a prognostic score of 0 to 2 were assigned to the low-risk group (n = 189), those with a score of 3 to 5 to the low-intermediate-risk group (n = 127), those with a score of 6 to 8 to the intermediate-risk group (n = 264), those with a score of 9 to 11 to the intermediate-high-risk group (n = 431), and those with a score of 12 to 13 to the high-risk group (n = 112). The survival curves according to the prognostic model are shown in Fig 1. There were significant survival differences among the five risk groups (*P* < 0.001). The 3-year survival rates for the low-, low-intermediate-, intermediate-, intermediate-high-, and high-risk groups were 98.9%, 92.8%, 82.4%, 58.4%, and 36.9%, respectively.

### Prediction of outcome of node-negative patients by the prognostic model

**Fig 2:**
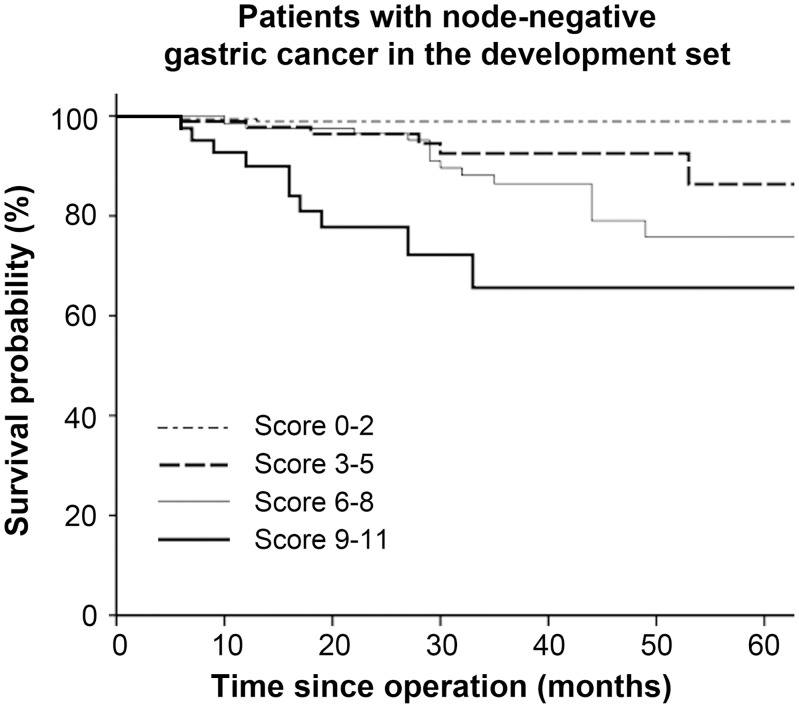
Survival curves based on risk groups for patients with node-negative gastric cancer in the development set (n = 440).

**Fig 3:**
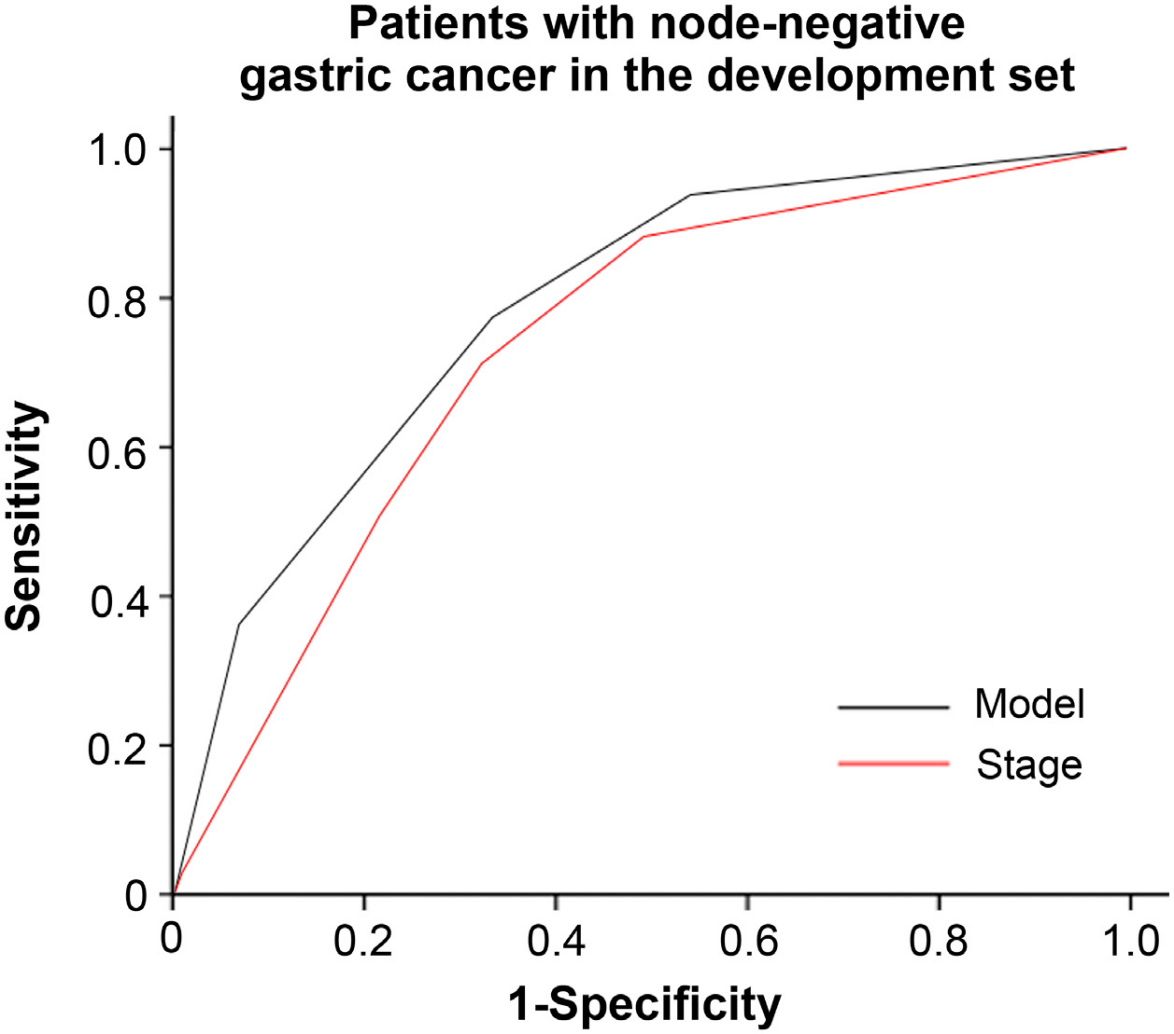
ROC plot of 3-year survival rate based on established model and TNM stage for patients with node-negative gastric cancer in the development set (n = 440).

Of all 1123 patients in the development set, 440 had node-negative gastric cancer. The prognostic model separated the patients without lymph node involvement into four risk groups (no patients had a score of 12–13) with significantly different survival outcomes (Fig 2). Among the 440 patients, 186 were assigned to the low-risk group, 90 to the low-intermediate-risk group, 123 to the intermediate-risk group, and 41 to the intermediate-high-risk group. Three-year survival rates for the low-, low-intermediate-, intermediate-, and intermediate-high-risk groups were 98.9%, 92.5%, 86.4%, and 65.6%, respectively (*P* < 0.001). The model yielded an area under the ROC curve of 0.78 for prediction of mortality at 3 years, which was superior to TNM staging with an area under the curve of 0.73 (Fig 3).

**Fig 4:**
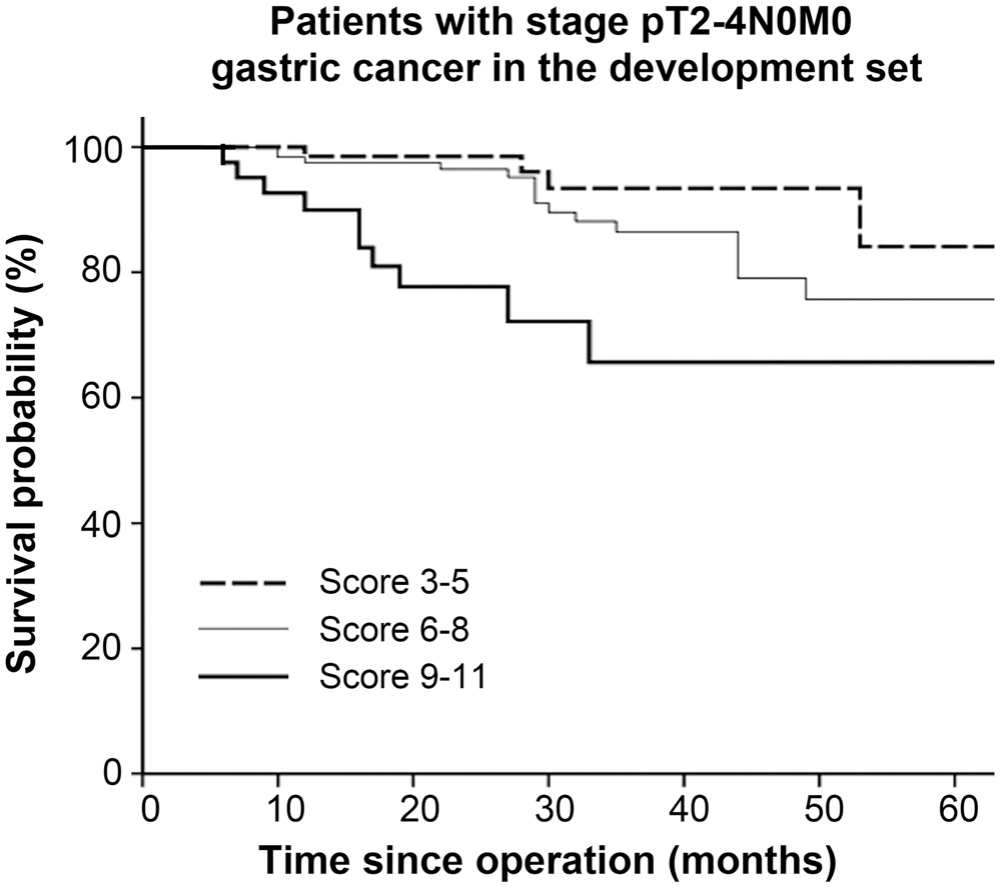
Survival curves based on risk groups for patients with stage pT2-4N0M0 cancer in the development set (n = 232).

**Fig 5:**
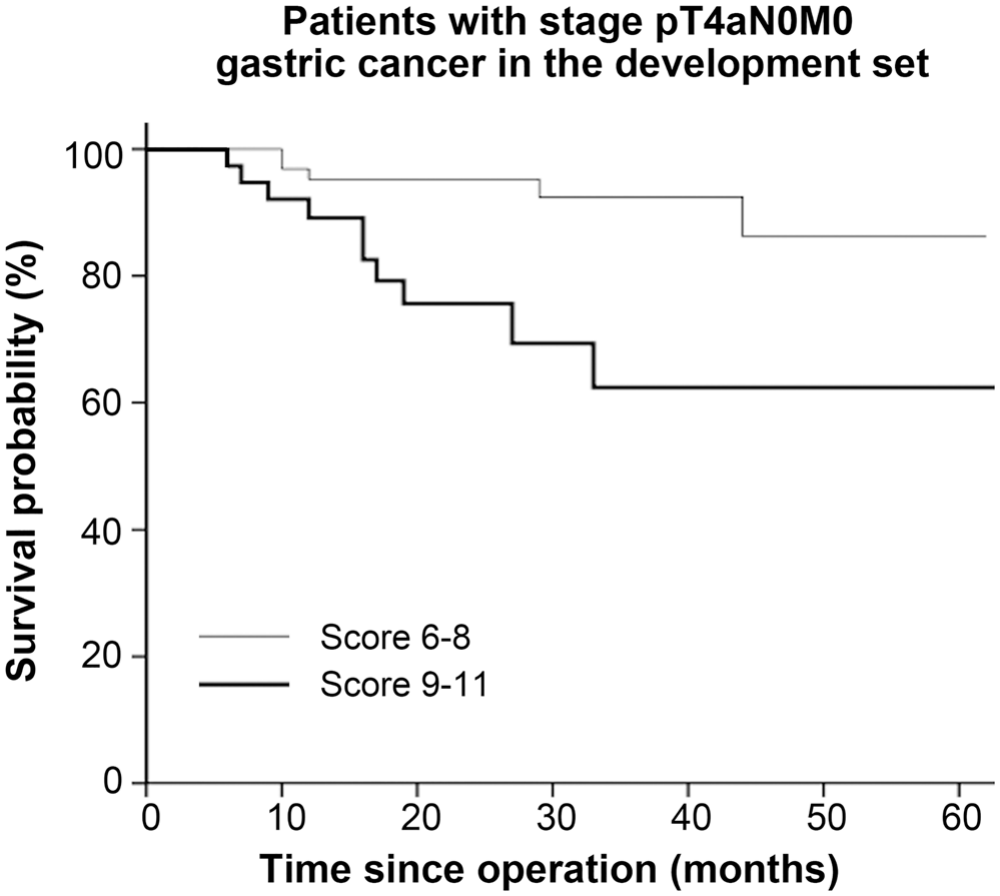
Survival curves based on risk groups for patients with stage pT4aN0M0 cancer in the development set (n = 102).

The prognostic model divided patients with node-negative advanced gastric cancer (T2–T4, n = 232) into three risk groups with significant survival differences (*P* = 0.001) (Fig 4). Of the 232 patients (stage T2-4N0M0), 102 (44%) patients had T4a tumor. The prognostic scores were then used to stratify the patients into two risk groups. Significant differences in the 3-year survival rate were observed between the two groups (92.5% vs 62.4%, *P* = 0.004) (Fig 5).

### Validation of the prognostic model

**Fig 6:**
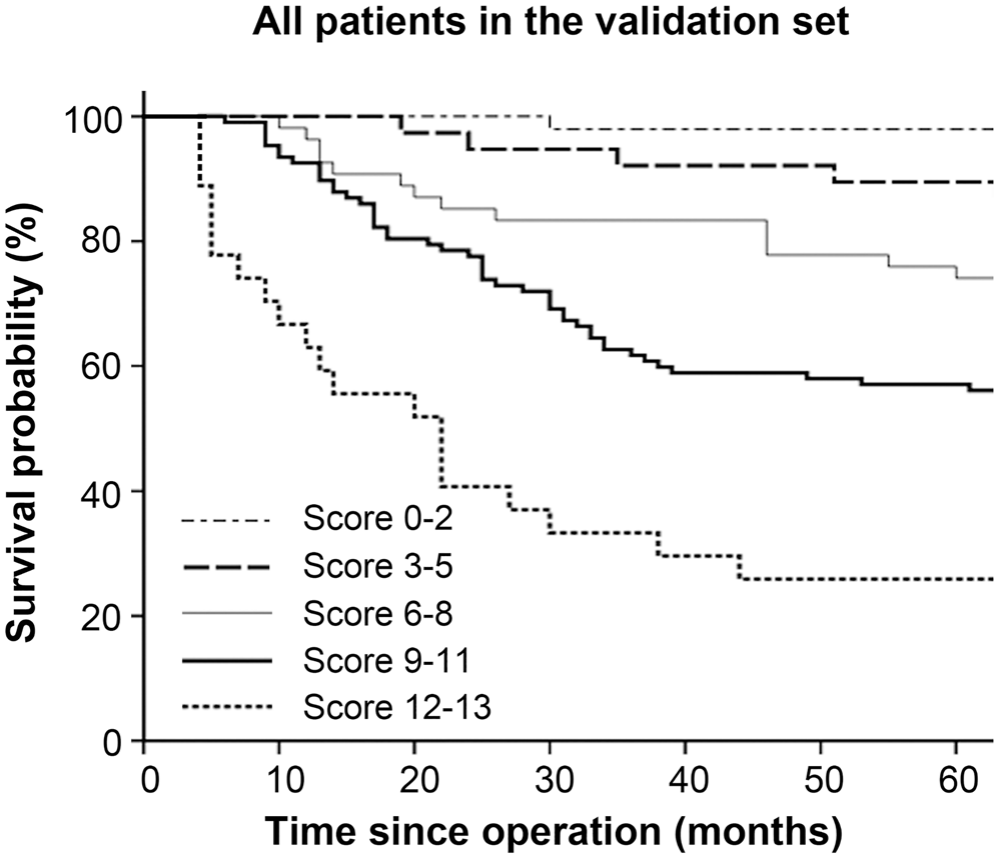
Survival curves based on risk groups for all patients who underwent gastrectomy and D2 lymphadenectomy in the validation set (n = 274).

We evaluated our prognostic model in an independent validation set of 274 patients. Using the scoring system, the proportions of patients classified into each risk category were similar. Among the 274 patients, 48 were assigned to the low-risk group, 38 to the low-intermediate-risk group, 54 to the intermediate-risk group, 107 to the intermediate-high-risk group, and 27 to the high-risk group. The survival curves according to the prognostic model are shown in Fig 6. Three-year survival rates for the low- to high-risk groups were 97.9%, 92.1%, 83.3%, 61.7%, and 33.3%, respectively (*P* < 0.001). Of all 274 patients, 105 had node-negative gastric cancer. The prognostic model separated the node-negative patients into four risk groups (no patients had a score of 12–13), and three-year survival rates for the low-, low-intermediate-, intermediate-, and intermediate-high-risk groups were 97.7%, 96.3%, 88.9%, and 62.5%, respectively (*P* = 0.005).

## Discussion

Prognostic models for patients with gastric cancer have been constructed before. Most studies included patients with stage I to IV disease or patients with metastatic/recurrent gastric cancer [14–16], only a few studies involved patients undergoing curative resection alone [17,18]. However, the prognostic factors were not consistent among patients undergoing radical gastrectomy, those undergoing palliative surgery, and those with inoperable disease; thus, different models should be used to predict outcomes in different groups of patients. Marrelli et al. used five variables (nodal status, depth of invasion, extent of lymphadenectomy, tumor location, and age) to predict the probability of recurrence in patients undergoing radical gastrectomy [17]. The model included no variables related to the host’s reaction to the tumor; however, such variables were recently reported to be associated with the prognosis of gastric cancer. Mohri et al. investigated the role of host- and tumor-related factors in predicting survival after curable gastrectomy [18]. This model, which was based on the NLR, tumor size, and clinical T grouping, offered a preoperative prediction of prognosis. However, the preoperative clinical TNM stage is estimated by radiological findings and is not in complete accordance with the postoperative pathological TNM stage. The reported accuracy of endoscopic ultrasound examination for T and N stage of tumor is 57% and 50%, respectively [19]. Therefore, a model based on postoperative pathologic staging would be more accurate than a model based on preoperative clinical staging. Additionally, recent studies have shown that systemic inflammatory response could be complementary to the TNM classification in predicting patients’ outcomes [9,20]. Therefore, in the present study, we constructed a prognostic model based on systemic inflammatory markers and clinicopathologic parameters for patients who underwent gastrectomy with D2 lymphadenectomy. The model separated patients into five different risk groups, among which the 3-year survival rates were significantly different. Furthermore, we externally validated our model in an independent cohort, finding that our model performed as well in the validation set as in the development set.

Whether adjuvant chemotherapy can improve survival for patients with node-negative gastric cancer remains controversial. The inconsistent results of clinical trials suggest that not all node-negative patients can benefit from adjuvant chemotherapy. Therefore, it is important to select patients by risk stratification to ensure tailored chemotherapy. Many recent studies have identified prognostic factors in patients without nodal involvement, such as depth of tumor invasion, lymphovascular invasion, and tumor size [21–23]. However, the prognostic significance of the systemic inflammatory response remains uncertain for these patients. Furthermore, Du et al. constructed a prognostic risk model of patients with pT2N0 gastric cancer based on lymphatic/blood vessel invasion, tumor diameter, and perineural invasion [24]. Nevertheless, a model applied to patients with stage pT1-4N0M0 tumor has not been proposed before. The current prognostic model based on all patients undergoing gastrectomy clearly discriminated patients with stage pT1-4N0M0 tumor into four different risk groups. The results indicated that the established model was suitable for all patients with resectable gastric cancer, whether or not associated with lymph node metastasis. We also assessed whether the model was associated with more accurate prognostic prediction for node-negative patients than was the pathological T stage. The results showed that the model increased the prediction accuracy of 3-year survival by 5.0%, indicating that the model plays a role complementary to that of traditional TNM classification. In the CLASSIC study, most patients without lymph node metastasis had serosal invasion [2], which is categorized as T4a tumor in the seventh edition of the AJCC TNM Staging System. The present prognostic model separated the patients with stage pT4aN0M0 tumor into two significantly different risk groups. Patients with higher scores had a poor 3-year survival rate (62.4%), and these patients might be likely to benefit from adjuvant chemotherapy. In contrast, patients with lower scores had a high 3-year survival rate (92.5%) and might not need chemotherapy, thus avoiding treatment-induced toxicity. Based on our data, patients in the high-risk group might be good candidates for adjuvant chemotherapy, and the model may be used to design clinical studies and explore therapies in defined sets of patients.

Our multivariate analysis showed that older age (≥65 years), larger tumors (>4.5 cm), diffuse or mixed type tumors, deeper tumor invasion, more lymph node metastasis, and a higher NLR were significant prognostic factors for poor survival in patients with resectable gastric cancer. Many recent studies have shown that the levels of systemic inflammatory markers such as C-reactive protein, albumin, fibrinogen, and circulating cellular components are useful prognostic markers for gastric cancer [10,20,25,26]. Our results demonstrated that among the examined factors accessible to clinicians, only a higher NLR was an independent predictor of mortality in patients with resectable gastric cancer. C-reactive protein was not incorporated in this study because it is not routinely examined as part of the preoperative evaluation. Our data are in accordance with a recent study that analyzed 357 patients with gastric cancer undergoing gastrectomy [18]. A high NLR is considered to reflect the host’s reaction to the biological behavior of the tumor. High numbers of neutrophils and/or low numbers of lymphocytes may promote tumor growth and metastasis or suppress lymphokine-activated killer cells, thereby counterweighing the antitumor immune response [10,11].

Although adjuvant chemotherapy can now improve the outcome of gastric cancer resection, the effect of adjuvant chemotherapy remains limited. Therefore, an accurate evaluation of prognosis is particularly important for identifying patients who may benefit from chemotherapy, sparing them from ineffective treatment. In the present analysis, patients with scores of 0 to 2 had a relatively higher 3-year OS rate (98.9%); these patients might not benefit from adjuvant chemotherapy, thus avoiding the toxicity of chemotherapy. For patients with a moderate risk of death, adjuvant fluorouracil monochemotherapy could be an option; S-1 monochemotherapy was more effective for early disease based on the subgroup analysis of the ACTS-GC study. Patients with high scores had a relatively poor prognosis, and intensive postoperative chemotherapy with multiple agents may be the optimal treatment strategy.

To the best of our knowledge, the present study is the first to delineate a convenient prognostic model incorporating readily available inflammatory markers and clinicopathologic parameters for patients undergoing potentially curative resection of gastric cancer. This prognostic model may assist clinicians in individual risk stratification, allowing more appropriate treatments for each patient, especially patients with node-negative gastric cancer. Based on our results, postoperative adjuvant chemotherapy may be optimal for node-negative patients with high risk. Nevertheless, definitive conclusions should not be drawn until prospective randomized controlled trials are performed. Further studies addressing treatment strategies based on risk stratification are warranted to maximize the efficacy of chemotherapy and reduce unnecessary chemotherapy.
